# ﻿Two novel *Tuber* species (Tuberaceae, Pezizales) from southwestern China based on morphological and molecular evidence

**DOI:** 10.3897/mycokeys.119.143714

**Published:** 2025-07-11

**Authors:** Tian-Jun Yuan, Hong-Mei Luo, Kai-Mei Su, Shu-Hong Li, E-Xian Li

**Affiliations:** 1 Biotechnology and Germplasm Resources Institute, Yunnan Academy of Agricultural Sciences, Kunming 650223, Yunnan, China Yunnan Academy of Agricultural Sciences Kunming China; 2 School of Science, Mae Fah Luang University, Chiang Rai 57100, Thailand Mae Fah Luang University Chiang Rai Thailand

**Keywords:** Ascomycetes, Latisporum group, new taxa, phylogenetic analysis, taxonomy

## Abstract

Based on morphological and molecular phylogenetic analyses, two new species, *Tubermarroninum* and *Tuberconditum*, belonging to the Latisporum group, were described from Yunnan province of southwest China. Morphologically, *Tubermarroninum* is distinguished from other Latisporum species by whitish and glabrous ascomata and a two-layered peridium, measuring 150–200 µm in total thickness, with an outer layer ranging from 40–60 µm. *Tuberconditum* is diagnosed by its thinner peridium (120–200 µm) and larger ascospores (49–66.5 × 34.5–60 μm in one-spored asci). According to the outcome of the ITS rDNA sequence analysis, the species of *T.marroninum* and *T.conditum* each form a distinct and well-supported group within the Latisporum group, respectively.

## ﻿Introduction

*Tuber* F.H. Wigg (Tuberaceae) is one of the most important genera in Pezizales in terms of both ecology and economy. It is widespread in Asia, Europe, and North America (Mello et al. 2006; Jeandroz et al. 2008; Trappe et al. 2009; García-Montero et al. 2010; Bonito et al. 2013; Zambonelli et al. 2016), with a few species also occurring in North Africa (Cacialli 2005). *Tuber* spp. establish obligate mycorrhizal symbiotic relationships with diverse plant hosts, such as oaks, pines, poplars, and commercially important trees, including chestnut, pecan, and hazelnut (Geng et al. 2009; Trappe et al. 2009; Wan et al. 2015). Not only can mycelium of *Tuber* spp. provide water and mineral nutrition for their host trees, but these fruiting bodies also contribute to the diet of small forest mammals. Some mycophagous animals are considered major consumers and vectors of hypogeous ectomycorrhizal fungi (Schickmann et al. 2012). Additionally, the fruiting bodies of some species, such as *Tubermagnatum*, *T.melanosporum*, *T.aestivum*, and *T.indicum*, have high value as a delicious food because of their unique scent. Although edible fungi are an important component of Chinese culture, there appear never to be accounts of *Tuber* species in ancient Chinese literature (Wang and Liu 2009). *Tubertaiyuanense* was the first reported species in 1985 in China (Liu 1985). Results of the last 20 years of research have revealed that China is a rich country in truffle diversity (Wang and Liu 2009). More than 60 truffle species have been documented in China, with the majority classified into the Puberulum, Latisporum, Maculatum, Rufum, *and*Melanosporum groups (http://www.speciesfungorum.org/Names/Names.asp). *Tuber* species in the Puberulum, Maculatum, and Gibbosum groups were commonly called “whitish truffle” to distinguish them from Italian white truffle, *T.magnatum* (Bonito et al. 2010; Lancellotti et al. 2016). The Rufum and Melanosporum groups are called “black truffle” (Bonito et al. 2010). In this study, two whitish new truffle species have been discovered in Yunnan province, China.

## ﻿Materials and methods

### ﻿Morphological studies

Fresh samples were collected from forests of *Pinusyunnanensis* Franch. in Yunnan, China. Microscopic and macroscopic characteristics are described based on the specimen materials (L3694 and L3385) following the methods of Yang and Zhang (2003). Color codes were given based on Kornerup and Wanscher (1978). Sections were made with a razor blade by hand, mounted in a 5% (w/v) KOH solution, then lactophenol cotton blue, and examined under an OLYMPUS BH-2 microscope. For scanning electron microscopy (SEM), ascospores were scraped from the dried gleba onto double-sided tape and mounted directly on an SEM stub. They were then coated with gold-palladium, examined, and photographed with a JEOL, JMS-5600LV SEM. For evaluation of the range of ascospore size, at least 40 ascospores each from one specimen of each collection site were measured. For ascospores, *Q*, the length-to-width ratio, is given in the same format as spore dimensions, and ***Q*** is the average *Q* of all specimens ± standard deviation. The specimens were deposited in the herbarium of the Biotechnology and Germplasm Resources Institute, Yunnan Academy of Agricultural Sciences (YAAS), and the Herbarium of Cryptogams, Kunming Institute of Botany, Chinese Academy of Sciences (HKAS).

### ﻿DNA extraction, PCR, and sequencing

Total DNA was extracted from pieces of dried ascomata with a modified CTAB methodology (Hofstetter et al. 2002, Li et al. 2011). Polymerase chain reactions (PCR) were performed using the primer combination ITS4/ITS5 (Hofstetter et al. 2002, Larsson and Sundberg 2011), nuc 28S rDNA subunit (28S) were amplified with primers LROR/LR5 (Vilgalys and Hester 1990), bRPB2-5F/bRPB2-7.1R (Matheny 2005) were used to amplify the second-largest subunits of RNA polymerase II (RPB2) (Sung et al. 2007) and 983F/2212R (Rehner and Buckley 2005) were amplified with primers translation elongation factor 1-α gene (tef-1α). In 25 µL of PCR reaction solution were contained 1 µL of DNA, 1 µL (5 µm) of each primer pair, 2.5 µL of 10 × buffer (Mg^2+^ plus), 1 µL of dNTP (1 mM), 0.5 µL of BSA (0.1%), 0.5 µL of MgCl_2_, and 1 U of Taq DNA polymerase (Takara Tag, Takara Biotechnology, Dalian, China). PCR reactions were run as follows: for the ITS gene, 94 °C for 5 min, followed by 35 cycles of 94 °C for 30 s, 54 °C for 1 min, and 72 °C for 1 min. The final reaction was followed by an extension at 72 °C for 10 min. For the LSU gene, 94 °C for 5 min, followed by 35 cycles of 94 °C for 1 min, 60 °C for 50 s, 72 °C for 1 min, and a final extension at 72 °C for 10 min. The PCR products were sent to Sangon Biotech Corporation (Shanghai, China) for purifying and sequencing by using ITS4/ITS5, LROR/LR5, bRPB2-5F/bRPB2-7.1R, and 983F/2212R, respectively.

### ﻿Phylogenetic analyses

**Figure 1.**
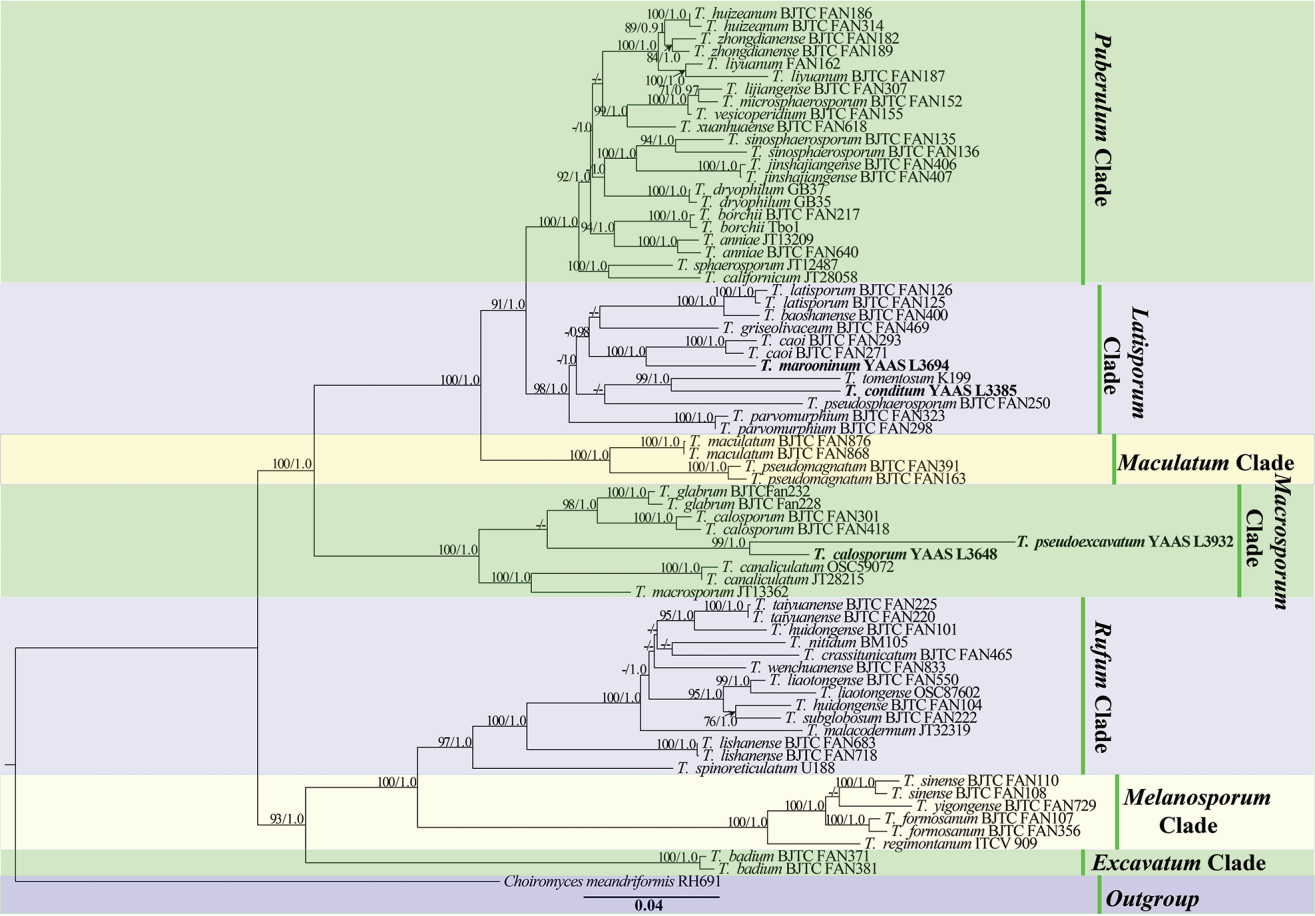
Phylogeny generated from maximum likelihood and Bayesian inference analysis of the ITS-LUS-TEF1-RPB2 rDNA sequences from *Tubermarroninum* and *T.conditum*-related species. *Choiromyceshelanshanensis* and *C.meandriformis* served as outgroups. ML bootstrap values (>75%) and Posterior Probabilities (PPs) values (>0.90) are shown above or beneath the branches at nodes. Novel sequences are printed in bold.

**Figure 2.**
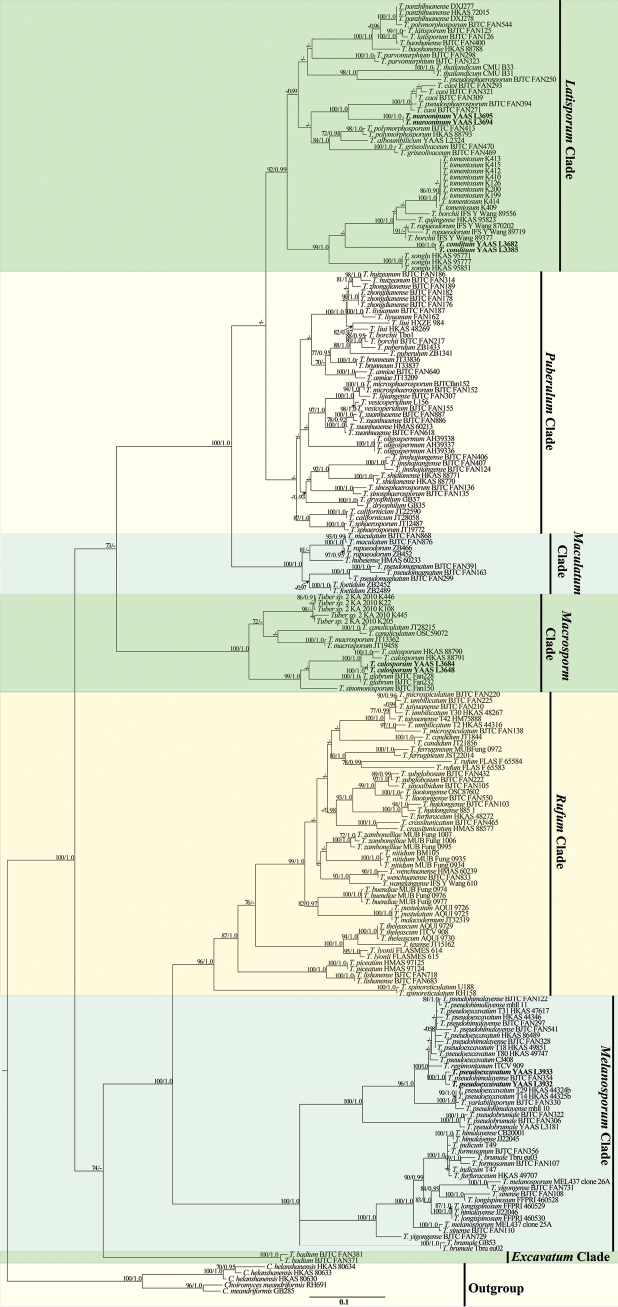
Phylogeny generated from maximum likelihood and Bayesian inference analysis of the ITS1-5.8S-ITS2 sequences from *Tubermarroninum* and *T.conditum* related species. *Choiromyceshelanshanensis* and *C.meandriformis* served as outgroups. ML bootstrap values (>75%) and Posterior Probabilities (PPs) values (>0.90) are shown above or beneath the branches at nodes. Novel sequences are printed in bold.

The *Tuber* sequences for phylogenetic analysis were obtained in this study and from the GenBank database. A total of 210 sequences (including 202 sequences by collections of *Tuber* and 8 sequences by sequencing in this study) (Table 1), in which 5 sequences derived from two species of *Choiromyceshelanshanensis* and *C.meandriformis* were selected and used as outgroups (Figs 1, 2). Sequences were edited and assembled using SeqMan Pro (DNAStar, Inc.). Alignment was performed using the online version of the multiple sequence alignment program MAFFT v7 (Katoh and Standley 2013), applying the G-INS-I strategy, and the alignments were manually adjusted in BioEdit. Poorly aligned sites were identified, and ambiguous sites were excluded by Gblocks v. 0.91b (Castresana 2000; using default options except “Allowed Gap Postions” = half) with default parameters. The multiple sequence alignments of both ITS-LSU-RPB2-TEF1 and ITS1 + 5.8S + ITS2 (based on the sequence of *Tuberglabrum*NR_153217, Fan et al. 2014) were carried out, establishing the phylogenetic trees, respectively. A maximum likehood (ML) phylogenetic tree was constructed using RAxML v7.0.3 on the online server accessed at https://mafft.cbrc.jp/alignment/server/, applying the rapid bootstrapping algorithm for 1000 replications using the GTRGAMMA model (Stamatakis et al. 2005; Stamatakis 2014). Clades with bootstrap values (BS) ≥ 75% were considered as significantly supported (Hillis and Bull 1993). Bayesian inference (BI) was performed using the Metropolis-coupled Markov chain Monte Carlo (MCMCMC) method in MrBayes version 3.2.7a (Ronquist et al. 2012), and the GTR + I + G model was selected as the best model under the Akaike Information Criterion (AIC) implemented by MrModeltest v.2.3 (Nylander 2004). Two independent runs of chains were conducted for 2,000,000 (ITS-nrLSU-RPB2-TEF1) and 1,000,000 (ITS1 + 5.8S + ITS2). The average standard deviations of split frequencies (ASDSF) were less than 0.01 at the end of the run, and ESS (effective sampling size) values were more than 200. Trees were sampled every 100 generations after burn-in (well after convergence), and 50% majority-rule consensus trees were constructed and viewed with TreeView32 (Page 2001). Bayesian posterior probabilities (PP) ≥ 0.90 were considered as significant support (Alfaro et al. 2003).

**Table 1.** Details of the *Tuber* sequences used in phylogenetic analysis. Sequences newly generated for this study are in bold.

Name	Voucher/isolate/strain	ITS	LSU	RPB2	TEF1
*C.helanshanensis*	HKAS_80633	KU531604	OM366182	OM584242	OM649586
*C.helanshanensis*	HKAS_80630	KU531603	OM366202	OM584265	OM649607
*C.helanshanensis*	HKAS_80634	NR_153899	OM366156	OM584208	OM649562
*Choiromycesmeandriformis*	RH691	HM485330	FJ809794	JQ954471	JX022550
*C.meandriformis*	GB285	HM485331	OM366158	OM584209	OM649563
*T.alboumbilicum*	YAAS_L2324	NR_155904	OM366174	OM584231	OM649578
*T.anniae*	JT13209	HM485338	JQ925680	/	JX022567
*T.anniae*	BJTC_FAN640	OM286868	OM366215	OM584274	OM649620
*T.baoshanense*	BJTC_FAN400	OM256791	OM366197	OM584260	OM649602
*T.baoshanense*	HKAS_88788	NR_178114	OM366176	OM584233	OM649580
*T.borchii*	IFS Y. Wang 89556	DQ478624	OM366222	OM584280	OM649629
*T.borchii*	IFS Y. Wang 89377	DQ478656	/	/	/
*T.badium*	BJTC FAN381	OM256748	OM366193	OM584256	OM649598
*T.badium*	BJTC FAN371	OM256747	OM366192	OM584255	OM649597
*T.borchii*	BJTC FAN217	KT067681	KT067706	OM584229	KT067717
*T.borchii*	Tbo1	MZ452886	/	MZ541897	MZ570608
*T.brumale*	GB53	FJ748900	/	/	/
*T.brumale*	Tbru-eu02	DQ329359	/	/	/
*T.brumale*	T.bru-eu03	DQ329360	/	/	/
*T.brunneum*	JT33836	KT897475	/	/	/
*T.brunneum*	JT33837	KT897479	/	/	/
*T.buendiae*	MUB_Fung-0974	NR_171295	/	/	/
*T.buendiae*	MUB_Fung-0976	MT006097	/	/	/
*T.buendiae*	MUB_Fung-0977	MT006098	/	/	/
*T.californicum*	JT28058	HM485346	JQ925685	JQ954496	JX022574
*T.californicum*	JT22590	HM485351	/	/	/
*T.calosporum*	HKAS_88791	KT444597	/	/	/
*T.calosporum*	HKAS_88790	KT444598	/	/	/
*T.canaliculatum*	OSC59072	HM485347	/	JQ954498	JX022576
*T.canaliculatum*	JT28215	JQ925643	/	JQ954497	JX022575
*T.candidum*	JT21856	HM485348	/	/	/
*T.candidum*	JT1844	HM485349	/	/	/
*T.caoi*	BJTC_FAN293	KP276182	KP276198	OM584240	KP276217
*T.caoi*	BJTC_FAN309	KP276181	/	/	/
*T.caoi*	BJTC_FAN321	KP276180	/	/	/
*T.caoi*	BJTC_FAN271	KP276183	NG_059984	OM584237	KP276216
*T.crassitunicatum*	BJTC_FAN465	MH115295	NG_088329	OM584268	OM649610
*T.crassitunicatum*	HMAS_88577	MH115297	/	/	/
*T.dryophilum*	GB35	JQ925644	JQ925687	/	JX022577
*T.dryophilum*	GB37	HM485354	JQ925688	JQ954501	JX022578
*T.ferrugineum*	JST22014	KX354297	/	/	/
*T.ferrugineum*	MUBFung-0972	MN962719	/	/	/
*T.foetidum*	ZB2452	JQ288905	/	/	/
*T.foetidum*	ZB2489	JQ288906	/	/	/
*T.formosanum*	BJTC_FAN107	MF621549	OM366159	OM584210	OM649564
*T.formosanum*	BJTC_FAN356	MF627986	OM366189	OM584252	OM649594
*T.furfuraceum*	HKAS_48272	GU979034	/	/	/
*T.furfuraceum*	HKAS_49707	GU979049	/	/	/
*T.glabrum*	BJTC_Fan228	NR_153217	NG_088326	OM584234	OM649581
*T.glabrum*	BJTC_Fan232	KF002727	OM366179	OM584236	/
*T.griseolivaceum*	BJTC_FAN469	KY428921	NG_088330	/	OM649612
*T.griseolivaceum*	BJTC_FAN470	KY428923	/	/	/
*T.himalayense*	CB20001	MW393547	/	/	/
*T.himalayense*	JJ22045	OQ054615	/	/	/
*T.himalayense*	JJ22046	OQ054619	/	/	/
*T.hubeiense*	HMAS_60233	KT067688	/	/	/
*T.huidongense*	BJTC_FAN103	MH115294	/	/	/
*T.huidongense*	885-1	MW201514	/	/	/
*T.huizeanum*	BJTC_FAN314	KT067685	KT067692	OM584246	KT067712
*T.huizeanum*	BJTC_FAN186	JQ910651	NG_059991	/	OM649575
*T.indicum*	T47	JQ639005	/	/	/
*T.indicum*	T49	JQ639007	/	/	/
*T.jinshajiangense*	BJTC_FAN124	KP276177	/	/	/
*T.jinshajiangense*	BJTC_FAN406	KX575841	OM366199	OM584262	OM649604
*T.jinshajiangense*	BJTC_FAN407	KX575842	OM366200	OM584263	OM649605
*T.latisporum*	BJTC_FAN126	KP276189	KP276204	OM584215	KP276205
*T.latisporum*	BJTC_FAN125	KT067676	KT067695	OM584214	KT067725
*T.liaotongense*	OSC87602	HM485369	FJ809813	/	JX022589
*T.liaotongense*	BJTC_FAN550	MH115302	OM366213	OM584272	OM649618
*T.lijiangense*	BJTC_FAN307	KP276188	KP276203	OM584244	KP276206
*T.lishanense*	BJTC_FAN683	MH115305	MH115306	OM584275	OM649621
*T.lishanense*	BJTC_FAN718	NR_160619	NG_064527	OM584276	OM649622
*T.liui*	HXZE_984	DQ478636	/	/	/
*T.liui*	HKAS_48269	DQ898182	/	/	/
*T.liyuanum*	FAN162	JQ771191.2	KT067698	OM584218	KT067710
*T.liyuanum*	BJTC_FAN187	JQ771193	KT067704	/	KT067719
*T.longispinosum*	FFPRI_460530	LC508589	/	/	/
*T.longispinosum*	FFPRI_460528	LC508587	/	/	/
*T.longispinosum*	FFPRI_460529	LC508588	/	/	/
*T.lyonii*	FLASMES-614	MT156453	/	/	/
*T.lyonii*	FLASMES-615	MT156454	/	/	/
*T.macrosporum*	JT13362	HM485373	FJ809838	/	JX022590
*T.macrosporum*	JT19458	HM485372	/	/	/
*T.maculatum*	BJTC_FAN868	OM265274	OM366227	OM584283	OM649634
*T.maculatum*	BJTC_FAN876	OM265278	OM366228	OM584284	OM649635
*T.malacodermum*	JT32319	FJ809889	JQ925702	JQ954514	JX022593
***T.marroninum***	**YAAS_L3694**	**ON454668**	**ON428904**	**OQ305202**	**OQ305199**
***T.marroninum***	**YAAS_L3695**	**OQ297680**	/	/	/
*T.melanosporum*	MEL437_clone_25A	EU555389	/	/	/
*T.melanosporum*	MEL437_clone_26A	EU555390	/	/	/
*T.microsphaerosporum*	BJTC_Fan152	KF805726	/	/	/
*T.microsphaerosporum*	BJTC_FAN152	KP276187	/	/	KP276207
*T.microspiculatum*	BJTC_FAN220	MH115315	/	/	/
*T.microspiculatum*	BJTC_FAN138	MH115317	/	/	/
*T.nitidum*	BM105	FJ809885	FJ809807	JQ954517	JX022597
*T.nitidum*	MUB:Fung-0934	MN962721	/	/	/
*T.nitidum*	MUB:Fung-0935	MN962722	/	/	/
*T.oligospermum*	AH39336	JN392267	/	/	/
*T.oligospermum*	AH39337	JN392268	/	/	/
*T.oligospermum*	AH39338	JN392266	/	/	/
*T.panzhihuanense*	DXJ277	JQ978651	/	/	/
*T.panzhihuanense*	DXJ278	JQ978652	/	/	/
*T.panzhihuanense*	HKAS_72015	NR_120126	/	/	/
*T.parvomurphium*	BJTC_FAN323	KP276185	KP276191	/	KP276215
*T.parvomurphium*	BJTC_FAN298	KP276186	NG_059981	OM584241	KP276214
*T.piceatum*	HMAS_97125	NR_160620	/	/	/
*T.piceatum*	HMAS_97124	MH115320	/	/	/
*T.polymorphosporum*	HKAS_88793	NR_178113	/	/	/
*T.polymorphosporum*	BJTC_FAN413	OM256794	/	/	/
*T.polymorphosporum*	BJTC_FAN544	OM256802	/	/	/
*T.pseudobrumale*	BJTC_FAN306	OM287838	/	/	/
*T.pseudobrumale*	BJTC_FAN322	OM287839	/	/	/
*T.pseudobrumale*	YAAS_L3181	KJ742703	/	/	/
*T.pseudoexcavatum*	CJ408	HM485381	/	/	/
*T.pseudoexcavatum*	T14_HKAS_44325b	GU979039	/	/	/
*T.pseudoexcavatum*	T29_HKAS_44324b	GU979040	/	/	/
*T.pseudoexcavatum*	HKAS_44346	GU979041	/	/	/
*T.pseudoexcavatum*	T18_HKAS_49851	GU979043	/	/	/
*T.pseudoexcavatum*	T31_HKAS_47617	GU979045	/	/	/
*T.pseudoexcavatum*	T80_HKAS_49747	GU979046	/	/	/
*T.pseudoexcavatum*	HKAS 86489	MG871289	/	/	/
*T.pseudohimalayense*	BJTC_FAN122	MF627983	/	/	/
*T.pseudohimalayense*	BJTC_FAN297	OM287837	/	/	/
*T.pseudohimalayense*	BJTC_FAN328	OM287840	/	/	/
*T.pseudohimalayense*	BJTC_FAN354	OM287844	/	/	/
*T.pseudohimalayense*	BJTC_FAN541	OM287847	/	/	/
*T.pseudohimalayense*	mhll-10	MT446223	/	/	/
*T.pseudohimalayense*	mhll-11	MT446224	/	/	/
*T.pseudomagnatum*	BJTC_FAN163	JQ771192	KP276192	OM584219	KP276208
*T.pseudomagnatum*	BJTC_FAN299	KP276184	/	/	/
*T.pseudomagnatum*	BJTC_FAN391	OM265244	OM366195	OM584258	OM649600
*T.pseudosphaerosporum*	BJTC_FAN250	NR_153229	NG_059982	/	OM649584
*T.pseudosphaerosporum*	BJTC_FAN394	OM256789	/	/	/
*T.puberulum*	ZB1341	JF261381	/	/	/
*T.puberulum*	ZB1433	JF261382	/	/	/
*T.pustulatum*	AQUI_9725	MK211278	/	/	/
*T.pustulatum*	AQUI_9726	MK211279	/	/	/
*T.rapaeodorum*	IFS Y. Wang 89719	DQ478651	/	/	/
*T.rapaeodorum*	IFS Y. Wang 870202	DQ478654	/	/	/
*T.qujingense*	HKAS 95823	KX904885	/	/	/
*T.rapaeodorum*	ZB452	JF261396	/	/	/
*T.rapaeodorum*	ZB466	JF261397	/	/	/
*T.regimontanum*	ITCV_909	EU375838	NG_059920	JQ954520	JX022600
*T.rufum*	FLAS-F-65583	MT374049	/	/	/
*T.rufum*	FLAS-F-65584	MT374050	/	/	/
*T.shidianense*	HKAS_88770	KT444595	/	/	/
*T.shidianense*	HKAS_88771	KT444596	/	/	/
*T.sinense*	BJTC_FAN108	MF627968	OM366160	OM584211	OM649565
*T.sinense*	BJTC_FAN110	MF627970	OM366161	OM584212	OM649566
*T.sinoalbidum*	BJTC_FAN105	MH115298	/	/	/
*T.sinomonosporum*	BJTC_Fan150	KF002729	/	/	/
*T.sinosphaerosporum*	BJTC_FAN136	JX092087	KP276196	/	KP276211
*T.sinosphaerosporum*	BJTC_FAN135	JX092086	NG_059983	OM584217	OM649569
*Tuber* sp. 2 *KA-2010*	K22	AB553345	/	/	/
*Tuber* sp. 2 *KA-2010*	K108	AB553349	/	/	/
*Tuber* sp. 2 *KA-2010*	K205	AB553353	/	/	/
*Tuber* sp. 2 *KA-2010*	K445	AB553366	/	/	/
*Tuber* sp. 2 *KA-2010*	K446	AB553367	/	/	/
*T.songlu*	HKAS_95771	KX904883	/	/	/
*T.songlu*	HKAS_95777	KX904884	/	/	/
*T.songlu*	HKAS_95851	KX904886	/	/	/
*T.sphaerosporum*	JT12487	FJ809853	FJ809805	/	JX022609
*T.sphaerosporum*	JT19772	FJ809854	/	/	/
***T.conditum***	**YAAS_L3385**	**ON454665**	**ON428901**	/	/
***T.conditum***	**YAAS_L3682**	**ON454667**	/	/	/
*T.spinoreticulatum*	RH158	GQ221454	/	/	/
*T.spinoreticulatum*	U188	FJ809884	FJ809815	JQ954527	JX022608
*T.subglobosum*	BJTC_FAN222	KF002728	OM366175	OM584232	OM649579
*T.subglobosum*	BJTC_FAN432	MH115323	/	/	/
*T.taiyuanense*	T42_HM75888	GU979033	/	/	/
*T.taiyuanense*	BJTC_FAN210	OM311199	/	/	/
*T.texense*	JT15162	HM485391	/	/	/
*T.thailandicum*	CMU-B31	MW470964	/	/	/
*T.thailandicum*	CMU-B33	MW470967	/	/	/
*T.theleascum*	AQUI_9729	MK211283	/	/	/
*T.theleascum*	AQUI_9730	MK211284	/	/	/
*T.theleascum*	ITCV_908	NR_164592	/	/	/
*T.tomentosum*	K126	AB553447	/	/	/
*T.tomentosum*	K200	AB553449	/	/	/
*T.tomentosum*	K409	AB553450	/	/	/
*T.tomentosum*	K410	AB553451	/	/	/
*T.tomentosum*	K412	AB553452	/	/	/
*T.tomentosum*	K413	AB553453	/	/	/
*T.tomentosum*	K414	AB553454	/	/	/
*T.tomentosum*	K415	AB553455	/	/	/
*T.tomentosum*	K199	AB553448	AB553521	AB553561	AB553541
***T.pseudoexcavatum***	**YAAS_L3932**	**ON454670**	/	/	**OQ305200**
***T.pseudoexcavatum***	**YAAS_L3933**	**OQ297681**	/	/	/
*T.umbilicatum*	T2_HKAS_44316	GU979031	/	/	/
*T.umbilicatum*	T30_HKAS_48267	GU979032	/	/	/
*T.umbilicatum*	BJTC_FAN225	MH115325	/	/	/
*T.variabilisporum*	BJTC_FAN330	OM287841	/	/	/
*T.vesicoperidium*	BJTC_FAN155	JQ690071	JQ690068	/	KP276212
*T.vesicoperidium*	L156	JQ690072	/	/	/
*T.wanglangense*	IFS Y.Wang_610	DQ478637	/	/	/
*T.wenchuanense*	HMAS_60239	JX267044	/	/	/
*T.wenchuanense*	BJTC_FAN833	OM311256	/	/	/
***T.calosporum***	**YAAS_L3648**	**ON454666**	**ON428902**	**OQ305201**	**OQ305198**
***T.calosporum***	**YAAS_L3684**	**OQ297679**	/	/	/
*T.xuanhuaense*	BJTC_FAN618	MK045627	OM366214	OM584273	OM649619
*T.xuanhuaense*	BJTC_FAN887	MK045644	/	/	/
*T.xuanhuaense*	BJTC_FAN886	MK045645	/	/	/
*T.xuanhuaense*	HMAS_60213	NR_147436	/	/	/
*T.yigongense*	BJTC_FAN731	MF663714	/	/	/
*T.yigongense*	BJTC_FAN729	MF663716	/	OM584277	OM649623
*T.zambonelliae*	MUB_Fung-1006	MW632954	/	/	/
*T.zambonelliae*	MUB_Fung-1007	MW632955	/	/	/
*T.zambonelliae*	MUB_Fung-0995	NR_174649	/	/	/
*T.zhongdianense*	BJTC_FAN176	KP276178	/	/	/
*T.zhongdianense*	BJTC_FAN178	KT067679	/	/	/
*T.zhongdianense*	BJTC_FAN182	KT067680	KT067702	/	KT067721
*T.zhongdianense*	BJTC_FAN189	KT067689	KT067705	/	KT067718

Note: The symbol “/” representative was not detected, and the black fonts showed the object of study in this paper.

## ﻿Results

### ﻿Molecular phylogenetics

The phylograms of ITS-nrLUS-RPB2-TEF1 (446 bp, 850 bp, 902 bp, and 814 bp, respectively) and ITS1 + 5.8S + ITS2 (446 bp) sequences are shown in Fig. 1, and Fig. 2, respectively. ML and BI analyses produced similar tree topologies, and only the tree generated from the ML analysis is shown. Both phylograms indicated that specimens of *T.marroninum* and *T.conditum* form a monophyletic clade, which was distinct from other *Tuber* species with high BS and PP support. Based on the ITS1 + 5.8S + ITS2 analysis (Fig. 2), *T.marroninum* and *T.conditum* stand with the Latisporum clade together with *Tubertomentosum*, *T.rapaeodorum*, *T.songlu*, *T.thailandicum*, *T.pseudosphaerosporum*, *T.polymorphosporum*, *T.panzhihuanense*, *T.lastisporum*, *T.baoshanense*, *T.parvomurphium*, *T.alboumbilicum*, *T.griseolivaceum*, and *T.caoi*, and the phylogenetic tree showed their strong bootstrap support values (BS = 100% and PP = 1.0). In this study, two additional specimens of *Tuberpseudoexcavatum* and *Tubercalosporum* were collected through morphological and molecular identification (BS = 100% and PP = 1.0) and preserved in the Herbarium of the Institute of Forestry and Soil Sciences (YAAS), implying these two species might be common in the southwest region of China.

### ﻿Taxonomy

#### ﻿ Tuber marroninum

Taxon classificationFungiPezizalesTuberaceae

T. J. Yuan, S. H. Li & X. H. Wang sp. nov.

9F5806F8-A461-50AC-9DC9-F9966E09CB4F

901881

Fig. 3a–g

##### Typification.

China • Yunnan Province: Xiangyun County (25°20'768"N, 100°43'34"E), in soil under mixed forest, with *Pinusyunnanensis* dominant, 20 Sep 2020, S. H. Li L3694 (holotype YAAS L3694, paratype YAAS L3695).

##### Gene sequences ex holotype.

ON454668 (ITS); ON428904 (LSU); OQ305202 (RPB2); OQ305199 (TEF1); ex paratype: OQ297680 (ITS).

##### Etymology.

“Marroninum” refers to the maroon color of the ascoma.

##### Diagnosis.

*Tubermarroninum* differs from other species by its whitish and glabrous ascomata and maroon color of the ascoma, globose and subglobose or broad ellipsoid ascospores, with greyish-white alveolate-reticulate ornamentation.

##### Description.

**Figure 3.**
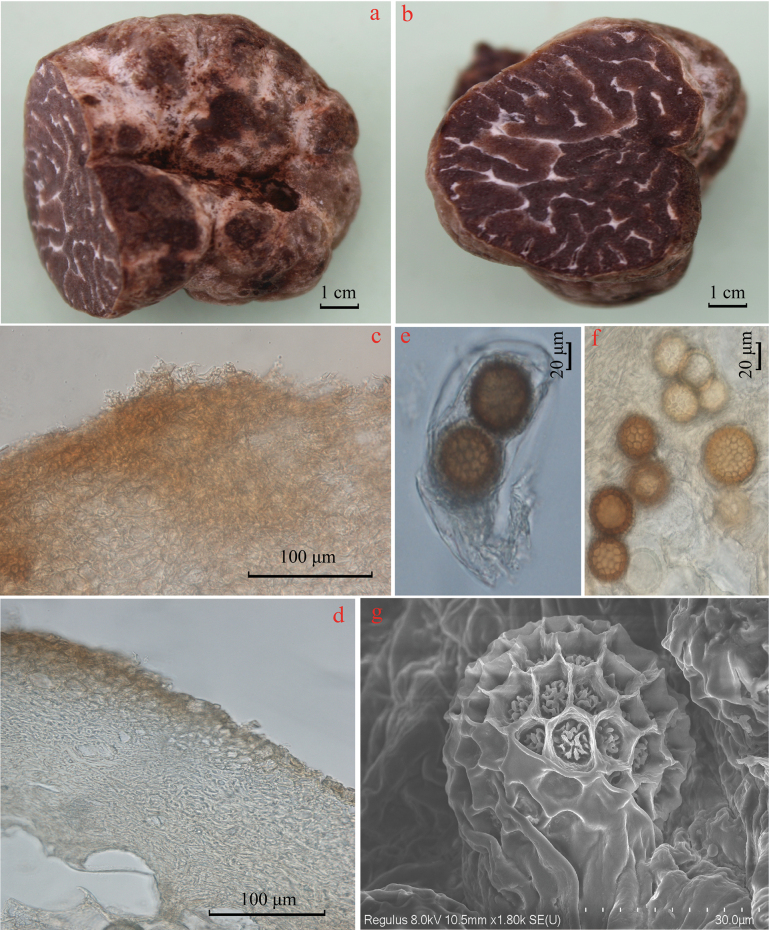
Photographs of *Tubermarroninum* (YAAS L3694, holotype): **a** fresh ascomata showing cut section **b** the gleba **c, d** vertical section of peridium **d** the gleba and asci **e, f** ascospores **g** scanning electron micrograph of ascospore.

***Ascomata*** (Fig. 3a, b) 3–5 cm diam, subglobose, hypogeous, whitish (3A1-2) becoming brown (8E6-8) when bruised, surface smooth, with white furrows; odor mild, taste not recorded. ***Peridium*** (Fig. 3c, d) two-layered, 150–200 µm thick, outer layer 40–60 µm thick, pseudoparenchymatous, composed of subglobose or irregularly shaped cells of 7.5–16.8 (24.5) µm broad with thickened walls, inner layer composed of thin-walled hyaline interwoven hyphae, 2.5–4 (5.5) µm diam, large cells of 20 × 10 µm diam. Sometimes, intermixed. ***Gleba*** (Fig. 3b) solid, firm, brown to dark brown (10F6-8) at maturity, marbled with whitish (1A1-1B1) narrow veins. ***Asci*** (Fig. 3e, g) subglobose or irregular, 1–4 spored, with a short stalk, 50–75 (100) × 40–55 (80) μm (n = 50). ***Ascospores*** (Fig. 3e, g) subglobose to globose, pale yellow (5A2-3, 5B3) when young, becoming brown (5C6-8, 5D6-7) at maturity, excluding their spiny-reticulate ornamentation, 41.5–52 × 41–50 μm, *Q* = 1.00–1.14 (n = 55), in one-spored asci, 33–41 × 32–40 μm (n = 50), *Q* = 1.00–1.15, in two-spored asci, 21–37 × 20–35.5 μm, *Q* = 1.01–1.12 (n = 60), in three-spored asci, 20–35.5 × 18.5–30.5 μm, *Q* = 1.02–1.15 (n = 60), in four-spored asci, *Q* = 1.14 ± 0.07, with a greyish white (2B1-2; 1C1-2) reticulatum and alveolate-reticulate ornamentation, meshes 5–8 across the ascospore width, 1.5–3 μm tall.

##### Distribution and habitat.

China: Yunnan province, Xiangyun county, hypogeous, in the soil in mixed woods dominated by *Pinusyunnanensis*.

##### Notes.

*Tubermarroninum* is phylogenetically closely related to *T.caoi* (Fan et al. 2016). In morphology, *T.caoi* has gray and pubescent ascomata, but *T.marroninum* has whitish and glabrous ascomata. Both trees of ML and BI (Figs 1, 2) also have strong bootstrap supporting the new species.

#### ﻿ Tuber conditum

Taxon classificationFungiPezizalesTuberaceae

T. J. Yuan, S. H. Li & X. H. Wang sp. nov.

297F5F49-CFC4-55A9-8BCA-6FBC2FB54C7E

901882

Fig. 4a–f

##### Typification.

China • Yunnan Province: Changning County (38.3903°N, 102.3535°E), in soil under mixed forest, with *Pinusyunnanensis* dominant, 20 Sep 2020, S. H. Li L3385 (holotype YAAS L3385, paratype YAAS L3682).

##### Gene sequences ex holotype.

ON454665 (ITS); ON428901 (LSU); ex paratype: ON454667 (ITS).

##### Etymology.

Conditum, Latin, refers to the acrid smell of ascocarp.

##### Diagnosis.

*Tuberconditum* differs from other species by having no cystidia on surface of ascomata, thinner peridium, and larger ellipsoid ascospores.

##### Description.

**Figure 4.**
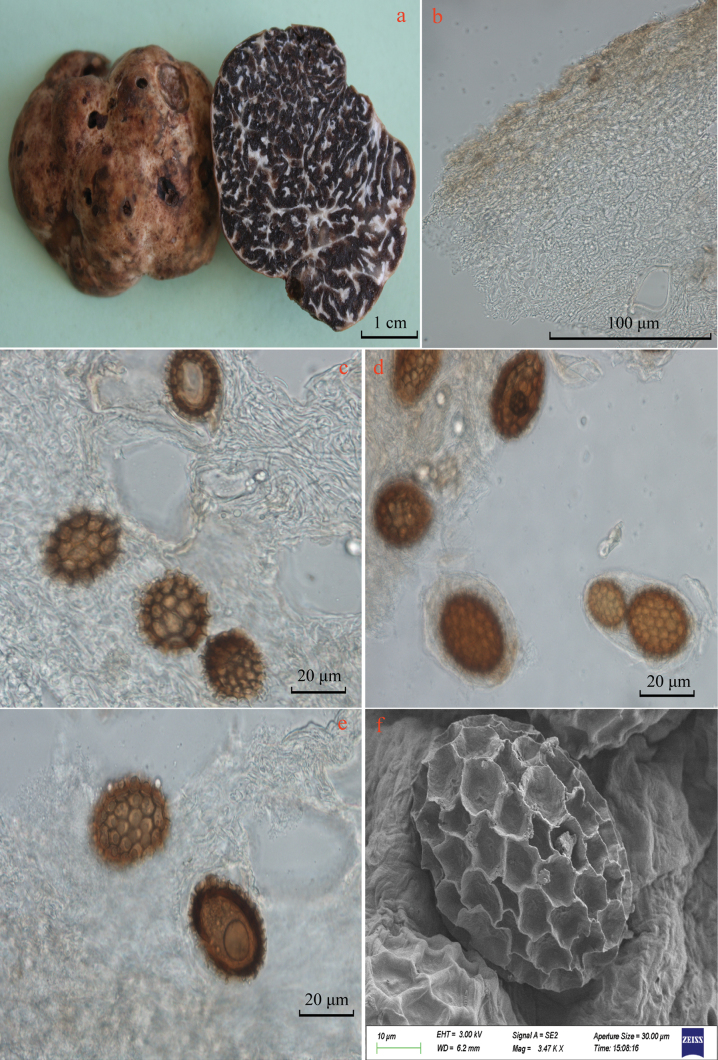
Photographs of *Tuberconditum* (YAAS L3385, holotype): **a** fresh ascomata showing cut section **b** vertical section of peridium **c, d** the gleba and asci **e** ascospores **f** scanning electron micrograph of ascospore.

***Ascomata*** (Fig. 4a) 3–4 cm diam., subglobose, hypogeous, grey-white to grey-brown (4A2-4; 4B3-4), surface smooth, with a few pinholes; acrid smell, taste not recorded. ***Peridium*** (Fig. 4b) one-layered, 120–200 µm thick, prosenchymatous, composed of interwoven hyphae 1.5–2.5 µm broad with thin-walled, and lumens 1.5–2.5 (3.5) µm diam, with ellipsoid or irregular cells of 15 × 10 μm diam. Sometimes intermixed, wall thickness 1–2 μm. ***Gleba*** (Fig. 4a, c) solid, firm, brown to black (6E7-8; (5-9)F8) at maturity, marbled with whitish narrow veins. ***Asci*** (Fig. 4d–e) subglobose or irregular, 1–4 spored, hyaline, thin-walled or occasionally with walls as thick as 2 μm, sessile stalk, 55–100 × 45–75 μm (n = 30). ***Ascospores*** (Fig. 4d–f) subglobose or ellipsoid, pale yellow (2A2-4), yellow-brown (5D6-8; 5E7-8) at maturity, excluding ornamentation, 49–66.5 × 34.5–60 μm, *Q* = 1.02–1.58 (n = 54), in one-spored asci, 33–54.5 × 30–47 μm, *Q* = 1.06–1.35 (n = 50), in two-spored asci, 21.5–44 × 21–37 μm, *Q* = 1.00–1.26 (n = 60), in three-spored asci, 20.5–40.0 × 20–35.5 μm, *Q* = 1.01–1.30 (n = 40), in four-spored asci, Q = 1.15 ± 0.23, reticulate ornamentation 2–5 μm in height, composed of irregular hexagonal meshes, 6–8 along the spore length and 3–6 along the breadth.

##### Distribution and habitat.

China: Yunnan province, Changning county, hypogeous, in the soil in woods dominated by *Pinusyunnanensis*.

##### Notes.

*Tuberconditum* is phylogenetically closely related to *Tubertomentosum* (Kinoshita et al. 2011), *T.qujingense*, and *T.songlu* (Wan et al. 2021). However, *T.conditum* has no cystidia on the surface of ascomata and larger ellipsoid ascospores, differing from *T.tomentosum* (Kinoshita et al. 2011; Sasaki et al. 2016). *Tuberconditum* differs from *T.qujingense* and *T.songlu* by its thinner peridium (120–200 µm) and larger ascospores (49–66.5 × 34.5–60 μm in one-spored asci). *Tuberqujingense* has a greyish-white ascomata, brown snowflake-shaped gleba, and prosenchymatous peridium, fusiform, and *T.songlu* has dense spine-like dermatocystidia, which are completely different from each other. In addition, DNA sequence analysis revealed the similarity between two species is less than 82.73% (*T.qujingense*) and 93% (*T.songlu*) in ITS sequences, strongly supporting the separation of *T.conditum* from the Chinese species of *T.qujingense* and *T.songlu.* Phylogenetic analysis (Figs 1, 2) also suggested that *T.conditum* is a distinct species because all sequences of this new species clustered within a strong bootstrap (BS ≥ 99% and PP = 1.0). The truffle has the acrid smell, which might be a good identifying feature for the fungus.

#### ﻿ Tuber pseudoexcavatum

Taxon classificationFungiPezizalesTuberaceae

Y. Wang et al., Cryptogamie Mycologie, 1998, 19(1–2): 113–120 (Manjón et al. 2009; Moreno et al. 1997)

26B2FE97-E50F-567B-8677-59AD4D2C13F4

##### Notes.

The description of *Tuberpseudoexcavatum* Y. Wang et al. (1998) was originally based on two specimens collected in China and published by Wang et al. in 1998. Subsequently, additional collections (AH 18387, AH 18331) from China were successfully used to generate DNA data, which were deposited in the Herbarium of the Institute of Forestry and Soil Sciences. This study reports two new collections that suggest the presence of this species may not be uncommon in the southwestern region of China. Morphologically, *T.pseudoexcavatum* is characterized by subglobose ascocarps with a deeply excavate appearance, ranging in color from brown to brown-orange. The surface exhibits a coarsely warted texture, while the asci contain 1–8 ascospores with spinoreticulate ornamentation, which may serve as diagnostic features based on the current collections. The known distribution of this species is limited to China.

##### Material examined.

Location • Yunnan Province, Changning County (37.1212°N, 101.7307°E). Habitat: Hypogeous, found in soil beneath a pure *Pinusarmandii* forest Altitude: Approximately 2500 m Collection Date: 20 Sep. 2020 Specimens: YAAS L3932, YAAS L3933 Collector: S. H. Li Repository: Herbarium of Institute of Forestry and Soil Sciences (YAAS).

#### ﻿ Tuber calosporum

Taxon classificationFungiPezizalesTuberaceae

S. P. Wan et al. Mycoscience, 2016, 57(6): 393–399

91284B8B-6374-55C5-8A7D-DBFA121F8D3E

##### Note.

*Tubercalosporum* S. P. Wan et al. was originally described based on four specimens collected from Huize County in Yunnan Province, as detailed by Wan et al. in 2016. The specimens (wsp352, wsp145, wsp186, and wsp382) provided successful DNA data. This study introduces two additional collections, suggesting a broader distribution of the species in the southwest region of China. Morphologically, *T.calosporum* is recognized by its brownish-yellow ascocarps with a verrucose surface, featuring superficial yellow furrows. The ascospores are characterized by their large, ellipsoid shape and shallow alveolar ornamentation, which can serve as diagnostic traits based on the present collections. The species is known to occur exclusively in China.

##### Material examined.

Location • Yunnan Province, Xiangyun County (25°39'43"N, 100°52'27"E) Habitat: Found in soil beneath a mixed forest dominated by Pinusyunnanensis Collection Date: 20 Sep 2020 Specimens: YAAS L3648, YAAS L3684 Collector: S. H. Li Repository: Herbarium of Institute of Forestry and Soil Sciences (YAAS).

## ﻿Discussion

The similarities of ITS sequences of *T.marroninum* and *T.conditum* to all described *Tuber* species are lower than 95.5% and 89.0%, respectively. In the genus *Tuber*, species delimitation based on 96% similarity in ITS has been suggested by previous studies (Kinoshita et al. 2011, 2021). Moreover, *T.marroninum* and *T.conditum* can be distinguished from other related species by morphology alone. In addition, phylogenetically, *T.marroninum* forms a distinct branch with high support (ITS 100% BS and 1.0 PP), and its sister species, *T.caoi*, can be distinguished by ITS-LUS-TEF1-RPB2 and ITS1 + 5.8S + ITS2 combined ML trees (Figs 1, 2). *Tuberconditum* can be distinguished from *T.tomentosum*, *T.rapaeodorum*, and *T.songlu* by the ML tree of ITS-LUS-TEF1-RPB2 (Fig. 1) and from *T.tomentosum* by the ITS1 + 5.8S + ITS2 combined ML tree (Fig. 2).

Morphologically, *T.marroninum* resembles *T.caoi*, *T.jinshajiangense* (Fan et al. 2016), and *T.shii* (Wang et al. 2016) of Chinese species with regard to its regularly globose ascospores with alveolar ornamentation. However, *T.caoi* and *T.jinshajiangense* have gray or pale gray and pubescent ascomata, but *T.marroninum* has whitish and glabrous ascomata. There are obvious differences between both *T.marroninum* and *T.shii* from their ascomata and gleba. *Tubershii* often has some superficial furrows, is pale grey-brown or pale brown, and has distinctive silver-grey tints ascomata, its gleba with brown at maturity (Wang et al. 2016), but *T.marroninum* has whitish and brown glabrous ascomata, and its gleba is brown to dark brown at maturity. Additionally, *T.marroninum* differs from *T.caoi* (37.5–40 μm in one-spored asci), *T.jinshajiangense* (35–37.5 μm in one-spored asci), and *T.shii* (29–51.5 μm in one-spored asci) by its larger ascospore (41.5–52 μm in one-spored asci). *Tuberconditum* differs from other species by its grey-white to grey-brown ascomata, thinner peridium (120–200 µm), and larger ascospores (49–66.5 × 34.5–60 μm in one-spored asci). The close relationship species of *T.tomentosum* has abundant spiny cystidia on the peridium surface (Kinoshita et al. 2011), and *T.pseudosphaerosporum* has 2–4.5 cm diam and white or whitish-yellow ascomata (Fan and Yue 2013). Furthermore, *T.marroninum* and *T.conditum* can remarkably identify other Chinese species of the Latisporum group, such as *T.alboumbilicum* (Li et al. 2014), *T.baoshanense* (Wan et al. 2017), *T.elevatireticulatum* (Lin et al. 2018), *T.griseolivaceum* (Huang et al. 2017), *T.huiliense*, *T.luyashanense*, *T.microcarpum* (Fan et al. 2022), and *T.panzhihuanense* (Deng et al. 2013), based on the color and size of ascomata. Of course, the two pairs of truffles are quite different because the phylogenetic tree places them in a different position by ITS-LUS-TEF1-RPB2 and ITS1 + 5.8S + ITS2 combined ML trees (Figs 1, 2).

In conclusion, we demonstrated that *T.marroninum* is a new species characterized by the maroon color of the ascoma, and *T.conditum* is a new species characterized by its smooth ascocarp surfaces and larger ellipsoid ascospores. Our findings of these species, *T.conditum*, *T.songlu*, *T.qujingense*, *T.rapaeodorum*, and *T.tomentosum*, are divided within the same group by their morphologic characteristics and phylogenetic relationships. In addition, *T.marroninum*, *T.pseudosphaerosporum*, and *T.caoi* are divided within the same group by their morphologic characteristics and phylogenetic relationships (Figs 1, 2). However, they were revealed to possibly belong to the Latisporum group (Wan et al. 2016, 2017; Fan et al. 2022) by the phylogenetic relationships through adding more DNA sequences in this study.

### ﻿Key to the species of the Latisporum group

**Table:** 

1	One-layer peridium	**2**
–	Two-layer peridium	**4**
2	Peridium prosenchymatous	**3**
–	Peridium pseudoparenchymatous	**5**
3	Irregular and lobed	***T.panzhihuanense* (Deng et al., 2013)**
–	Surface smooth and with a few pinholes	***T.conditum***
4	Peridium smooth and glabrous	**6**
–	Peridium puberulent	**7**
5	Ascomata white umbilicate	***T.alboumbilicum* (Li et al. 2014)**
–	Ascomata whitish, becoming brown when bruised with white furrow	***T.marroninum***
6	Ascomata whitish-yellow and mostly much lobed with deep furrows	***T.pseudosphaerosporum* (Fan & Yue, 2013)**
–	Ascomata pale yellow to desert yellow with distinctively hygrophanous patch around or near the base	***T.parvomurphium* (Fan et al., 2016)**
7	Ascospores mainly globose	**8**
–	Ascospores mainly broadly ellipsoid	**9**
8	Ascospores globose only	***T.caoi* (Fan et al., 2016)**
–	Ascospores fusiform, globose or sometimes subglobose	***T.polymorphosporum* (Wan et al., 2017)**
9	Ascomata olive gray or gray with green	***T.griseolivaceum* (Huang et al., 2017)**
–	Ascomata pale yellowish brown or reddish brown	**10**
10	Ascospores broad ellipsoid	**11**
–	Ascospores ellipsoid, subglobose	**12**
11	Asci 2–3 spored, ascospores smaller (25–40 × 14–25 μm)	***Tubertomentosum* (Kinoshita et al., 2011)**
–	Asci 1–4 spored, ascospores larger (30–60 × 25–50 μm)	***Tuber* sp. 12 (Kinoshita et al., 2011)**
12	Ascomata ocher-yellow	**13**
–	Ascomata whitish	**14**
13	Asci 2–3 spored, ascospores smaller (30–35 × 20–30 μm)	***Tuber* sp. 11 (Kinoshita et al., 2011)**
–	Asci 2–3 spored, ascospores largerer (30–55 × 30–40 μm)	***Tuber* sp. 13 (Kinoshita et al., 2011)**
14	Ascospores reddish-brown, with five-spored in asci and alveolar walls up to 2–6 μm tall	***T.baoshanense* (Wan et al., 2017)**
–	Ascospores yellowish brown to brown, with four-spored in asci and alveolate reticulum 3–5 μm tall	***T.thailandicum* (Suwannarach et al., 2015)**
–	Ascospores yellowish brown to reddish brown, with four-spored in asci and alveolar walls up to 3–5 μm deep	***T.latisporum* (Chen & Liu, 2007)**

## Supplementary Material

XML Treatment for Tuber marroninum

XML Treatment for Tuber conditum

XML Treatment for Tuber pseudoexcavatum

XML Treatment for Tuber calosporum

## ﻿Additional information

### Data availability

All of the data that support the findings of this study are available in the main text.
